# Keeping Food on the Table: Human Responses and Changing Coastal Fisheries in Solomon Islands

**DOI:** 10.1371/journal.pone.0130800

**Published:** 2015-07-09

**Authors:** Simon Albert, Shankar Aswani, Paul L. Fisher, Joelle Albert

**Affiliations:** 1 School of Civil Engineering, the University of Queensland, Brisbane, Queensland, Australia; 2 Department of Anthropology, Rhodes University, Grahamstown, South Africa; 3 Department of Ichthyology and Fisheries Science, Rhodes University, Grahamstown, South Africa; 4 WorldFish, Honiara, Solomon Islands; University of California Santa Cruz, UNITED STATES

## Abstract

Globally the majority of commercial fisheries have experienced dramatic declines in stock and catch. Likewise, projections for many subsistence fisheries in the tropics indicate a dramatic decline is looming in the coming decades. In the Pacific Islands coastal fisheries provide basic subsistence needs for millions of people. A decline in fish catch would therefore have profound impacts on the health and livelihoods of these coastal communities. Given the decrease in local catch rates reported for many coastal communities in the Pacific, it is important to understand if fishers have responded to ecological change (either by expanding their fishing range and/or increasing their fishing effort), and if so, to evaluate the costs or benefits of these responses. We compare data from fish catches in 1995 and 2011 from a rural coastal community in Solomon Islands to examine the potentially changing coastal reef fishery at these time points. In particular we found changes in preferred fishing locations, fishing methodology and catch composition between these data sets. The results indicate that despite changes in catch rates (catch per unit effort) between data collected in 2011 and 16 years previously, the study community was able to increase gross catches through visiting fishing sites further away, diversifying fishing methods and targeting pelagic species through trolling. Such insight into local-scale responses to changing resources and/or fisheries development will help scientists and policy makers throughout the Pacific region in managing the region’s fisheries in the future.

## Introduction

A large proportion of the world’s fish stocks are believed to be either overfished, under-pressure or significantly depleted from historical over fishing [1–3]. Subsequently, there is rising concern over future food and nutrition security, particularly in developing countries where population growth is high and fish are the primary animal source protein [4].

The Pacific region has some of the highest global population growth rates, greatest consumption of fish per capita and limited access to arable lands [5,6]. This demographic process, coupled with external pressures (e.g. climate change and ineffective governance) is expected to lead to a shortfall in the ability of reef fisheries to supply the protein needs for the populations of several Pacific Island countries and territories, by 2030 [5–8].

Offshore and coastal fisheries are integral for the economies and food security in the Pacific. Offshore tuna fisheries are important for economic development (contributing up to 10% of the regions gross domestic product [9]), while coastal fisheries are critical for food security and rural incomes [10]. Recent assessments of key offshore tuna stocks (bigeye, yellowfin and skipjack) in the Central and Western Pacific suggest that stocks of skipjack and yellowfin tuna remain healthy, while stocks of bigeye tuna are under-pressure and require management intervention [11].

In contrast to offshore fisheries, coastal fisheries are relatively understudied and are data deficient [9,10]. There is limited information on catch and productivity, primarily due to the difficulty in data collection for this primarily subsistence-based fishery [12]. However, there is some information available on commercially valuable species, such as bêch-de-mer, trochus, giant clams and green snail which have shown dramatic decline and local extinctions in some parts of the region [10,13].

To date, estimates of fishing pressure and yield assessments have generally been conducted at regional or national scales. The general consensus being that population growth will soon outpace the fisheries ability to provide protein for many Pacific countries [6,14]. Results from village scale studies are mixed, with one instance in Fiji revealing that catch per unit effort has increased over time [15] as a result of urban drift. However, in other cases fishers haven’t observed changes in fisheries over recent decades [16]. On the other-hand, in Solomon Islands, some species of fish targeted for market, have showed indications of being overfished [17–19]. Understanding village scale responses to these potential shortfalls in fish based protein and overfishing of certain species is fundamental to provide insight into possible management actions that maintain food and nutrition security for rural Pacific communities.

Despite the warning of a looming shortfall in the supply of fish for millions of people in the Pacific, there is limited information on long-term changes in fish catches and stocks [9]. This is particularly true for Solomon Islands, where a comprehensive review of Pacific Island fisheries presented comparatively less data for Solomon Islands compared to other Pacific Island nations, which on the whole are considered to possess little data compared to other global regions [20,21]. Despite this, it has been suggested that Solomon Islands will require an additional fish catch of 18,750 tonnes p.a. by 2030 [6] to meet the country’s fish consumption demand, which is more than double the current catch rate (11,150 tonnes p.a.).

Fisheries in Solomon Islands, as with the majority of the Pacific Islands, are largely subsistence based multi-species reef-based fisheries with as little as 20% of the fish and invertebrate catch being sold at cash markets [22]. Due to the subsistence nature of fisheries, and widespread geographic setting of the Solomon Islands archipelago, there is little documentation of catch composition and the majority of fisheries are not subject to formal markets and record keeping. In addition, fishers return to numerous different fishing nodes making creel surveys by third party government or research organisations difficult. These limitations in record keeping are exacerbated when combined with the high diversity of species typically caught by subsistence fisheries, for example, reports of 100–200 species are not uncommon for reef fishers [14].

In this study we analyse the spatial and temporal characteristics of fishing behaviour of Baraulu fishers in the Roviana Lagoon, Solomon Islands, and compare fish catch data from 1995 to 2011 to examine changes to the coastal reef fishery between these time points. The key questions explored through this research were, given ongoing environmental changes [23,24], have fishers responded to ecological change by expanding their fishing range and/or effort? Answering these questions is fundamental for understanding human responses to resource scarcity in a rapidly changing environment. This paper provides a unique insight into local-scale responses to resource depletion that is relevant throughout the Pacific region.

## Materials and Methods

This paper focuses on data collected over two time periods, 1995 and 2011. In 1995 raw data were collected as part of a study on customary sea tenure and artisanal fishing within the Roviana Lagoon [25], which utilized voluntary self-reporting diaries and focal follows to obtain catch data. The work in 2011 was part of a broader national level study in partnership with the Solomon Islands Ministry of Fisheries and Marine Resources. As part of this national study, a self-reporting diary was developed for fishers to record information about their fishing catches. Village fishers were trained in record keeping. Using these two data sets, we analyzed changes in fish catch composition, location and methodology. Given that fishing technology and local generalized foraging methods/strategies have remained fairly constant since 1994, the methodology used in both surveys furnished data that are comparable.

### Ethics statement

In 1995, fish catch data was obtained on a voluntary basis and consisted solely of fisheries data provided by consenting individuals. In 2011, research clearance was provided through a memorandum of understanding that WorldFish has with the Solomon Islands Government and adhered to the WorldFish Code of Ethics for working with people (2009). Fishers involved in the study were informed on the purpose of data collection prior to research activities being initiated. Fisher consent was documented through self-reporting diaries; those not willing to participate did not provide information on their fishing activities. To maintain confidentiality of fishers, the information obtained through the self-reporting diaries and focal follows did not record personal information and was limited to information about their fishing activities.

### Study site

Baraulu is a rural coastal village of 400 people in the Roviana Lagoon, Western Province, Solomon Islands, S1 Fig. The lagoon is 400 km^2^, comprised of extensive shallow coral reefs, seagrass and mangrove habitat that supports a human population of over 15,000. While the general population has increased across the Solomon Islands since 1995, the population of Baraulu has remained fairly constant over this period as a result of rural-to-urban migration and a recent religious split that has divided the village into 2 different hamlets. For instance, a 1994 population census of the village registered 394 inhabitants (212 males/282 females), while a population census conducted in 2001 provided a population of 419 inhabitants (230 males/189 females), indicating a fairly stable population throughout a period of 7 years. The main Baraulu community is located on a raised barrier island on the southern side of the lagoon, adjacent to a passage connecting the lagoon to the open ocean. The adjacent lagoon is formed by a gradient of marine ecosystems including mangrove forests, river mouths, mudflats, seagrass, lagoonal reefs, barrier reefs, marine lakes, amongst others, and has characteristics of both coastal and coral atoll lagoons. The lagoon passages are wide and deep allowing for the exchange of water between the open ocean and the lagoon. The lagoon hydrodynamics has allowed for the development of diverse ecological communities, particularly coral reef communities of diverse ecological characteristics in the entrances and central zones of the lagoon. Beyond the passages is the open sea, which offers fishers the possibility of exploiting a number of deep sea coral reef species as well as pelagic fish such as tuna. Baraulu people have lived a largely subsistence lifestyle of small scale farming and fishing, yet today, their livelihood activities are threatening coral reefs as well as other marine and terrestrial habitats. Damaging activities include the small-scale, non-regulated exploitation of commercial species like holothurians, trochus, and various shell species, the increasing pressures on the subsistence fishery include small-scale commercial netting of fish, night diving for scarids (done primarily by external poachers) and for rock lobsters for the growing tourist industry, the collection of corals for building structures such as wharfs, the aquarium fish collection trade, and poor land based practices which cause sedimentation impacts on lagoon nursery areas [26]. This coupled with environmental effects related to climate change is increasingly degrading coral reefs and their future role in providing ecosystem services.

### Fish catch data

Between March and August in 1995 and 2011 fishers from Baraulu village collected information on their fishing trips. Approximately 50 individual fishers returned data in both 1995 and 2011 from a total of approximately 65 fishers in Baraulu village. In 1995 fishers were randomly selected throughout the week and in 2011 all fisher catches were documented on one day (Saturday) of each week. The following parameters were quantified for each fishing trip in both 1995 and 2011: number of fishers, paddling time, fishing time at each spot, fishing methods used, species caught, fish weight at each location, fishing location and habitat type, and distance from village. Diary entries were co-ordinated and checked for anomalous entries by a trained research assistant. Manual spring scales (± 50 g) were used for fish weight. Fish species were recorded in diaries in local vernacular in which fishers have a detailed taxonomic knowledge, which were identified to species or family level with fishers prior to data entry. For the purpose of data analysis, fishing methods were grouped into broad groupings of: trolling (nylon line, lure and hook towed behind a wooden paddle canoe), handline (nylon line (typically 10–30 lb) with baited hooks near bottom or non-baited hooks striking to attract and catch fish in mid-water), dropline (fishing with a lured hook using a stone as a weight, striking the stone off in mid-water), net (fishing with a net from either a canoe or from shore where people chase fish into the net), poison (using *Derris sp*. leaf to temporarily stun fish) and spear (freediving with wooden spear gun or sling).

In 1995 two research methods were employed: focal follows and self-reporting diaries. Focal follows were initially used in 1995 to optimize the use of self-reporting diaries and to ensure catch data were being reported correctly. Once confident that the diaries were being used correctly, diaries were the predominant reporting method. Statistical analysis of catch data from focal follow and diaries showed no significant difference for catch rate between methods (CPUE, ANOVA F_(1, 2378)_ = 2.4041, p = 0.12). Diary holders were recruited randomly to keep records of their fishing activities. The data collected during 1995 and 2011 were used to explore the effects of fishing method and fishing location/habitat type on mean net return rates and fishing event duration (see [27] for further discussion).

### Data analysis

All fishing trips involving harvesting of invertebrates were removed from the analysis due to high variability in fishing time and inability to compare invertebrate and fish harvest weights. In total, 767 fishing trips (over 895 fisher hours) were analysed in 1995 and 259 fishing trips (over 851 fisher hours) in 2011. The statistical software Statistica Ver. 12 (Statsoft, USA) was used to analyse for significant differences between data recorded in 1995 and 2011 using a Kruskal-Wallis ANOVA both for the entire data set and for each grouped fishing methods. Geographic information system maps were produced using ArcMap Ver. 10.2 (Esri, USA). Data is presented as mean ± standard error throughout the manuscript unless otherwise stated.

## Results

In both 1995 and 2011 fishers spent most of their time using handline, although handline use was higher in 1995 at 69.7% compared to 46.2% in 2011 (Table 1). Likewise the proportion of time spent net fishing was higher in 1995 (15.1%) compared to 2011 (6.7%). Conversely, the proportion of time spent dropline fishing was substantially lower in 1995 at 21.4% compared to 30.1% in 2011), as was trolling (10.6% and 15.3%, in 1995 to 2011 respectively), Table 1.

**Table 1:** Time spent fishing using various methods in 1995 and 2011 within the Roviana lagoon study area.

Fishing Method	1995		2011	
	Time Fishing (h)	% Total	Time Fishing (h)	% Total
Dropline	21.4	2.4	255.9	30.1
Handline	623.9	69.7	392.7	46.2
Net	134.9	15.1	57.0	6.7
Trolling	94.9	10.6	130.5	15.3
Poison	9.6	1.1	6.5	0.8
Spear	9.9	1.1	8.0	0.9
Total	894.5	-	850.5	-

**Fig 1:**
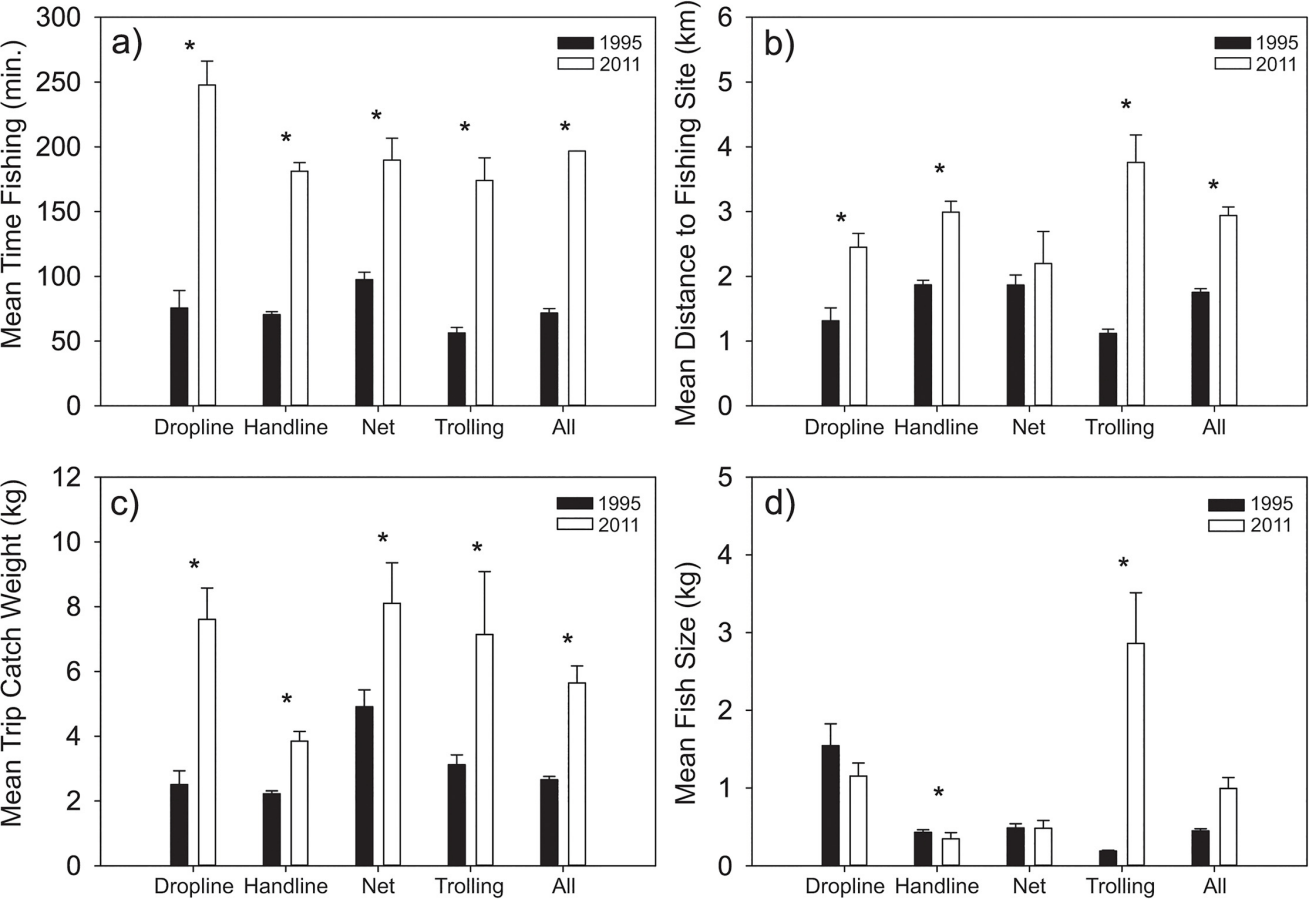
Catch parameters for the Roviana lagoon study area for data collected in 1995 and 2011. Panels show a) Mean time spent fishing in 1995 and 2011, b) mean distance to fishing site, c) mean fishing trip catch weight, d) mean fish size. Data significantly different between 1995 and 2011 using a Kruskal-Wallis test are shown (*). Statistical results for all fishing methods are shown in results section. Statistical results for individual methods are shown within S1 Table.

**Fig 2:**
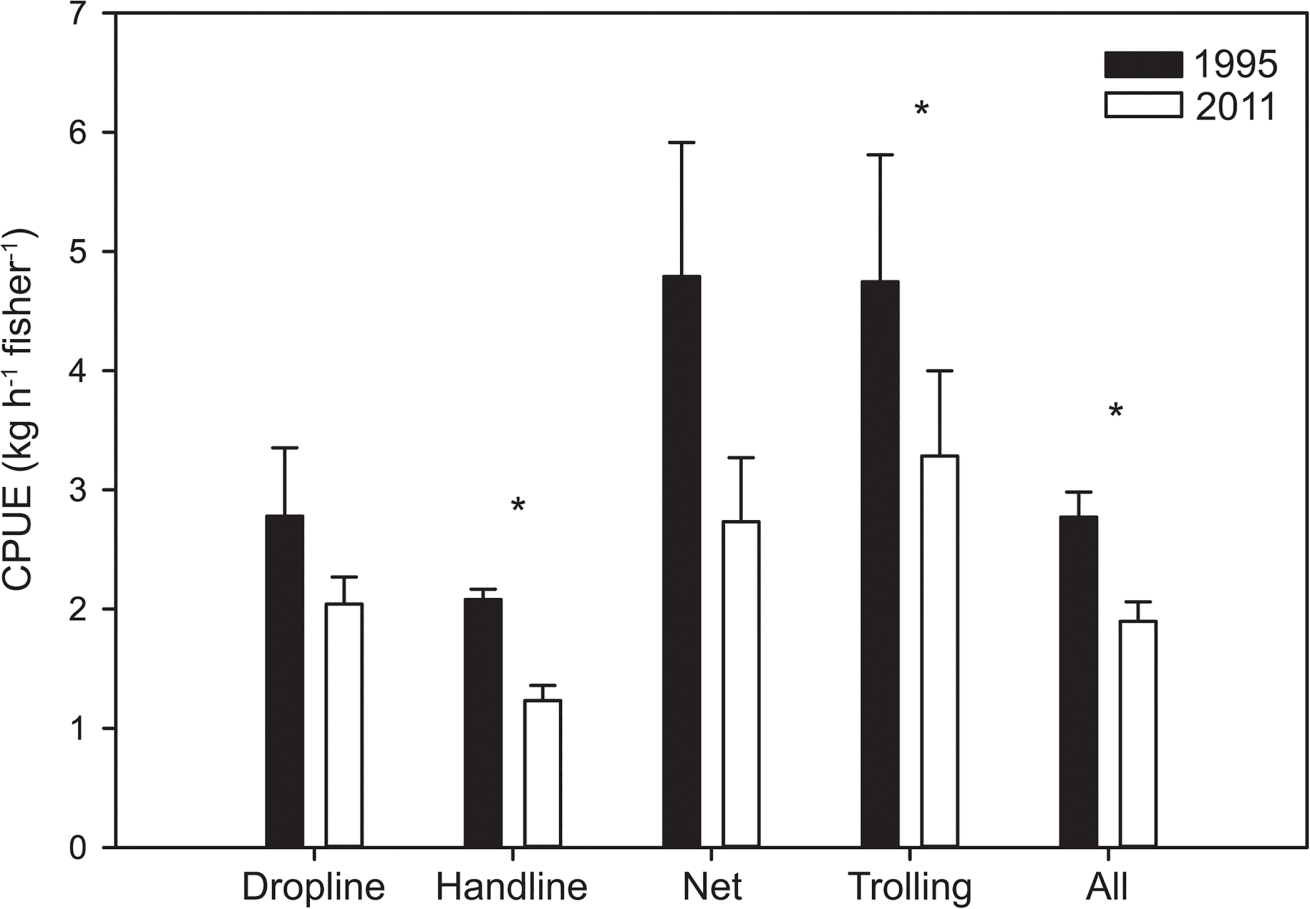
Catch per unit effort within the Roviana lagoon study area in 1995 and 2011. Graph indicates catch per unit effort (CPUE) within the study site for 1995 and 2011 by fishing method. Data significantly different between 1995 and 2011 using a Kruskal-Wallis test are shown (*).

The mean time spent fishing (excluding travel time) on each fishing trip was 71.7 (±3.76) minutes in 1995, while significantly higher at 197 (±0.04) minutes in 2011 (KW_H(1,988)_ = 358.67, p<0.001) (Fig 1A). Fishers travelled further to fish in 2011 (2.94 ±0.13 km) compared to 1995 (1.75 ±0.06 km) (KW_H(1,988)_ = 75.03, p<0.001) (Fig 1B). The average total weight of fish caught per trip was twice as high in 2011 compared to 1995 (5.6 ±0.52 kg and 2.7 ±0.10 kg, respectively (KW_H(1,988)_ = 358.67, p<0.001)) (Fig 1C), as was the mean weight of individual fish (0.99 ±0.14 kg and 0.45 ±0.03 kg, respectively). The difference in weights between the two years sampled was synergistic with an increased proportion of larger fish from trolling (0.19 ±0.01 vs. 2.86 ±0.65 kg) in 2011 (KW_H(1,145)_ = 6.98, p = 0.008) (Fig 1C). Whereas, the weight of fish caught by hand line showed the opposite trend (0.43 ±0.03 vs. 0.35 ±0.08 kg for 1995 and 2011). Overall fish catch rates, as measured using catch per unit effort (CPUE) was significantly higher in 1995 at 2.77 ±0.21 kg fisher^-1^ hr^-1^ compared to 1.90 ±0.16 kg fisher^-1^ hr^-1^ in 2011 (KW_H(1,988)_ = 24.86, p<0.001) (Fig 2). Similarly, catch rate for handline fishing alone were higher at 2.08 ±0.09 kg fisher^-1^ hr^-1^ in 1995 versus 1.23 ±0.13 kg fisher^-1^ hr^-1^ in 2011 (KW_H(1,662)_ = 30.89, p<0.001).

A diverse assemblage of fish were recorded in 1995 and 2011, including at least 90 species from 19 families. Species from the Carangidae family comprised the highest proportion of catch in both 1995 (34.8%) and 2011 (31.4%). Species from the Lutjanidae family were the second most frequently caught fish in 1995 (17.6%) however Lutjanids contributed to only to 7.7% of fish caught in 2011. The change in catch composition was compensated by an increased contribution of Scombridae to the catch from 1.4% in 1995 to 11.4% in 2011 (Table 2).

**Table 2:** Proportion of fish catch (abundance) by family and fishing methods for 1995 and 2011 within the Roviana lagoon study area.

Family	Proportion of Catch (%)									
	1995					2011				
	Dropline	Trolling	Net	Handline	All Methods	Dropline	Trolling	Net	Handline	All Methods
Acanthuridae	0.0	0.0	0.0	0.5	0.1	0.0	0.0	0.0	0.0	0.0
Balistidae	0.0	0.0	0.0	3.6	0.9	0.0	0.0	0.0	8.5	2.2
Carangidae	50.0	39.1	42.3	4.8	34.8*	32.7	56.1	24.5	6.9	31.4*
Chaetodontidae	0.0	0.0	0.0	0.0	0.0	0.0	0.0	0.0	0.3	0.1
Chanidae	0.0	0.0	0.0	0.0	0.0	0.0	0.0	4.1	0.0	1.1
Clupeidae	0.0	6.5	0.7	0.9	2.1	0.0	0.0	18.4	0.3	4.9
Haemulidae	0.0	0.0	0.0	0.0	0.0	0.0	0.0	0.0	0.6	0.2
Holocentridae	4.2	8.3	0.0	2.2	3.7*	0.0	1.2	0.0	0.6	0.5
Labridae	0.0	0.0	0.7	5.7	1.6	0.9	0.0	0.0	5.0	1.6
Lethrinidae	12.5	0.0	5.4	35.3	13.6*	2.7	1.2	0.0	42.0	12.0*
Lutjanidae	25.0	11.2	7.4	25.3	17.6*	5.5	0.0	4.1	19.9	7.7*
Monodactylidae	0.0	0.0	0.0	0.0	0.0	20.0	0.0	8.2	1.6	7.8*
Mullidae	0.0	0.0	34.2	0.5	8.9	0.0	0.0	22.4	0.3	5.9
Nemipteridae	0.0	0.0	0.0	7.9	2.0	0.0	0.0	0.0	0.6	0.2
Scaridae	0.0	0.0	0.0	0.1	0.0	0.0	0.0	2.0	0.0	0.5
Scombridae	4.2	1.2	0.0	0.0	1.4	4.5	30.5	8.2	0.3	11.4*
Serranidae	4.2	0.0	0.0	9.8	3.6	2.7	0.0	0.0	10.4	3.4
Sphyraenidae	0.0	29.6	6.0	2.7	9.8*	21.8	7.3	4.1	1.6	9.1
Teraponidae	0.0	0.0	0.0	0.0	0.0	0.9	0.0	0.0	0.0	0.2
Other	0.0	4.1	3.3	0.7	13.5	8.3	3.7	4.0	1.1	7.6

* Denotes highest five ranked families for “All Methods” for 1995 and 2011

**Fig 3:**
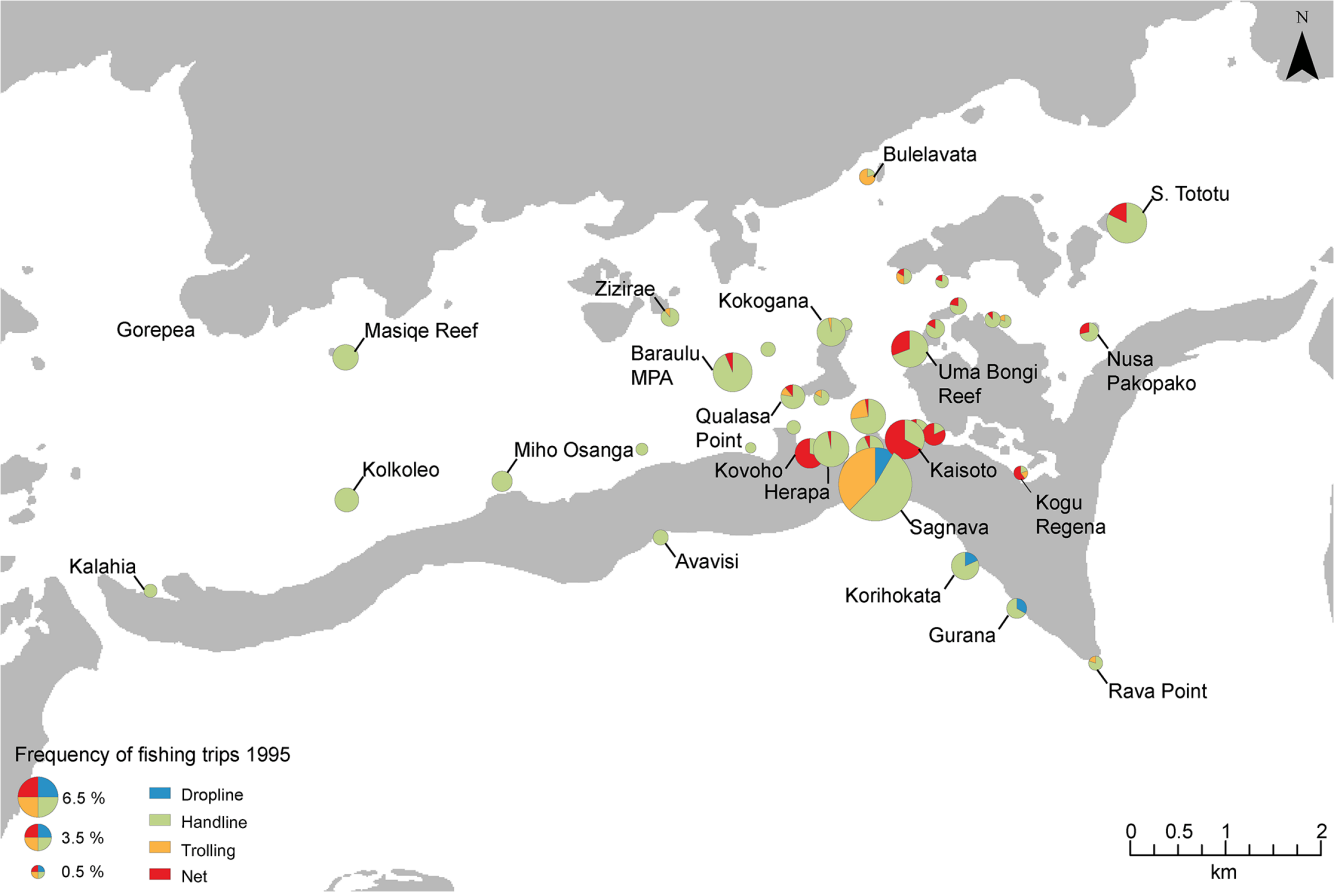
Proportion of fishing time and methods spent across fishing sites within the Roviana lagoon study area in 1995. Size of pie chart symbol denotes the the anmount of time spent at fishing locations throughout the Roviana lagoon during the 1995 record period. The amount of time spent for each method is shown by pie chart sections.

**Fig 4:**
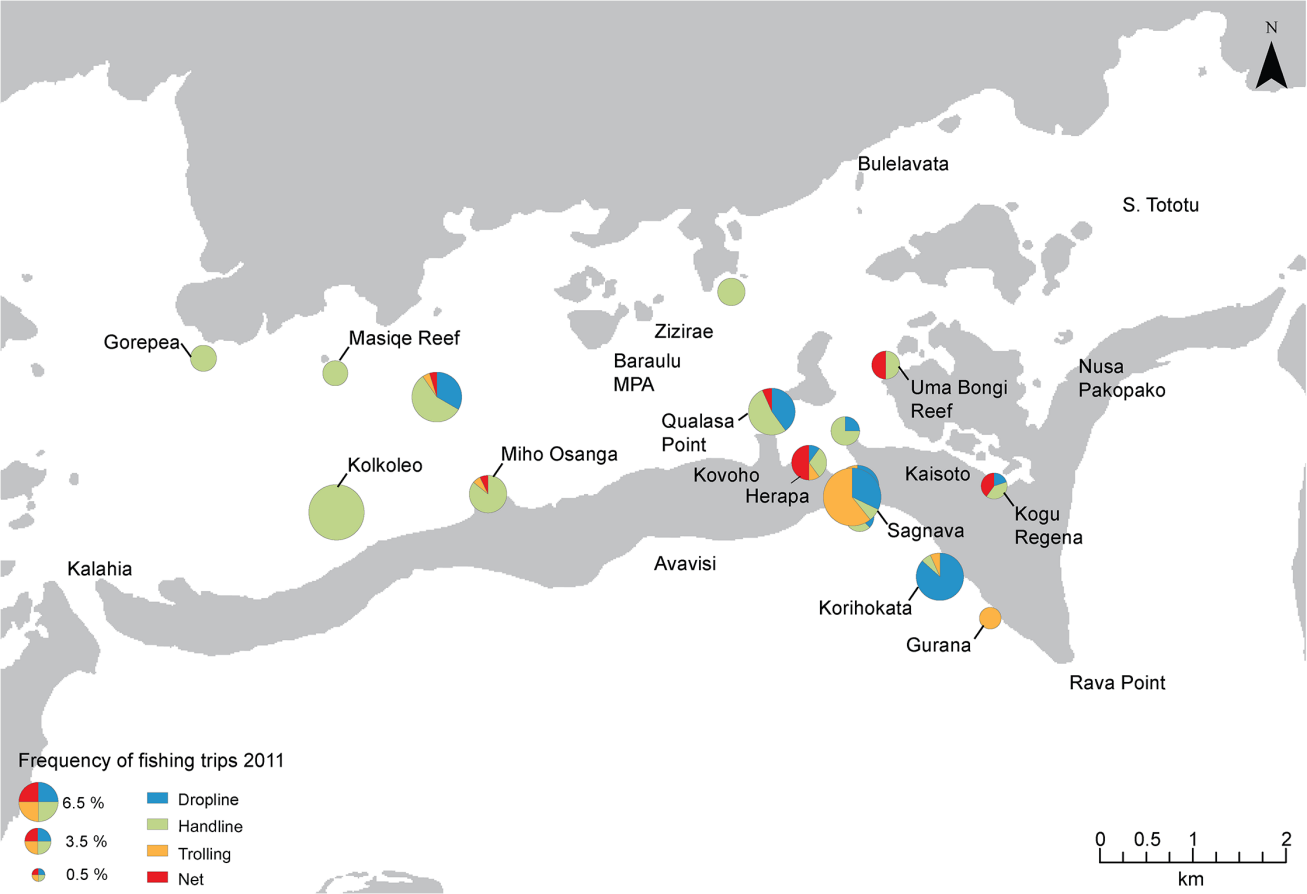
Proportion of fishing time and methods spent across fishing sites within the Roviana lagoon study area in 2011. Size of pie chart symbol denotes the the anmount of time spent at fishing locations throughout the Roviana lagoon during the 2011 record period. The amount of time spent for each method is shown by pie chart sections.

**Fig 5:**
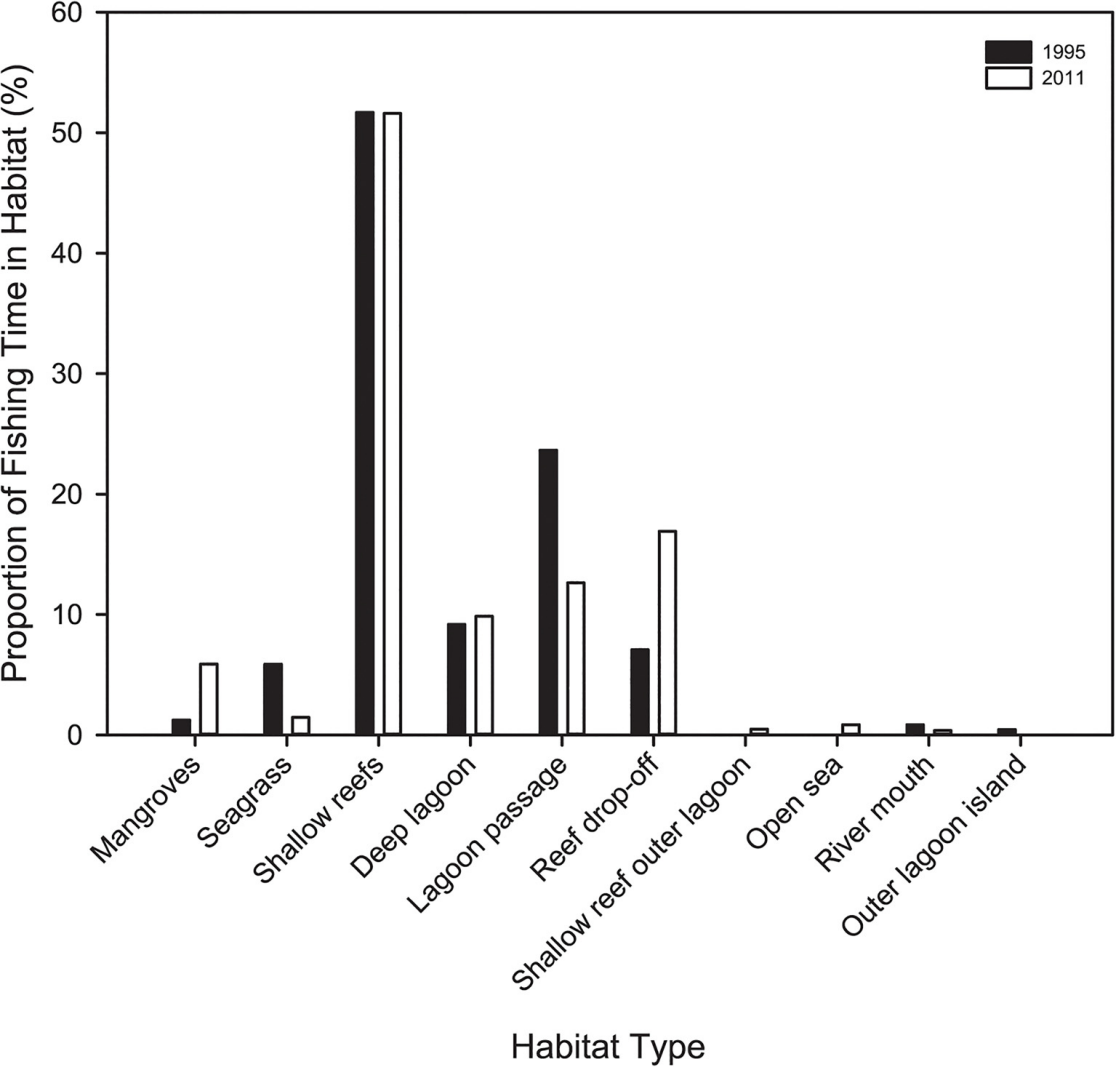
Proportion of fishing time spent within various habitats for 1995 and 2011 within the Roviana lagoon study area. Data for 1995 and 2011 are shown by black and white bars, respectively.

In 1995 fishers concentrated their fishing effort close to the village, using predominately handline angling (Fig 3). In 2011 fishing there was increased effort spread further from the village at fewer locations. In general, fishing methods were more diverse in 2011 than 1995, with a shift to more trolling in reef drop-off locations, such as Sagnava, Gurana and Korihokata (Fig 4). Fishers spent most of their time fishing in shallow reef habitat (51%) in both 1995 and 2011. Time spent fishing mangroves and reef drop-offs habitats was higher in 2011 whereas effort in seagrass and reef passages was lower (Fig 5).

## Discussion

Coastal fisheries play a fundamental role in supporting the subsistence livelihoods of millions of people globally. The remoteness of many subsistence communities on the small islands of the Pacific limits their ability to access alternative protein sources and hence fisheries productivity is a matter of basic survival [4]. This renders the predictions of imminent coastal fisheries decline across the Pacific [6,8] of particular concern. Solomon Islands are considered one of the more vulnerable nations with high population growth rate, high fisheries dependency and low GDP limiting access to other protein options [5]. However experience from large scale environmental disasters that have severely impacted fisheries in Solomon Islands suggests rural communities in Solomon Islands possess substantial adaptive capacity [23,28,29].

In order to adequately plan for the predicted shortfall in protein supply for millions of people across the Pacific an understanding of how fishers respond to changing fisheries productivity over time is essential. This study has clearly demonstrated that for the primary fishing method of handlining significantly lower catch rates (CPUE) were found in 2011 compared to data collected 16 years prior, with similar results observed for other fishing methods. This lower catch rate was coupled with a decrease in average fish size. Although several factors, other than reducing fish stocks, can result in reduced catch rates the results from this study concur with several other studies [30] and reinforce the notion that coastal fisheries may be in decline [31]. However, we are not able to completely exclude other factors such as socio-economic changes, external poaching, or change in fish catchability or aggregation. Indeed, the overall quantity of fish returned to the village on each fishing trip using handline increased substantially from 2.22 (±0.09) in 1995 to 3.8 (±0.30) kg per trip in 2011. This counter-intuitive increase was driven by both longer fishing times and travelling to fishing grounds further away. In 1995 the majority of the handline fishing effort was focussed within 2 km of the village, by 2011 this had increased to 3 km. So whilst fishers appear to have maintained and actually increased the quantity of fish returned to the village using handline, this was at a cost of paddling further and spending longer at fishing sites increasing fishing effort for individual fishers. Maintaining handline catches through paddling further may only provide a short term solution if fisheries are indeed declining in Roviana as customary fishing grounds of the Baraulu community only extend 3–4 km to the east and west and the current fishing effort is located on the extremities of these fishing grounds. In addition, the social impacts of this increased effort and time away from the village could be significant in the rural villages, as the fishers also have import roles to play in the household and gardens. As mentioned previously lower catch rates may also have resulted from an increase in population within the region between 1995 and 2011 resulting in an increase overall fishing effort for the region. National census data shows an annual growth rate of 2.6% for the Roviana Lagoon region between 1999 and 2009 [32], however, data available for Baraulu suggests the village population has remained relatively stable over time. Changes to target species between 1995 and 2011 may also have affected catch rates if new target species have a lower catchability. However this would appear not to be the case in Baraulu, with Lethrinidae and Lutjanidae dominating the handline catch in both years (Table 2).

The higher number of fish being returned to the village in 2011 was also supported by a substantially higher proportion of fishing time spent both trolling and droplining in 2011. These methods may have offset the reduced effort spent on handlining in fishing grounds close to the village. Despite the lower trolling catch rates recorded in 2011 there was a clear increase in fish size, distance travelled to fishing grounds, time spent fishing, and the average catch weight from paddle canoe based trolling. The increase in fish size and catch was driven by an increase in Scombridae and Carangidae. This trend may point towards a process where declining handlining of reef fish from shallow coral reefs could be supplemented through trolling for pelagics in deeper waters providing the potential for a longer term solution to a potential fisheries crisis. Whilst the tuna resource in the Western Pacific is under pressure from commercial operations, it is still one of the most intact fisheries globally and diversifying access to tuna is promoted as a means to increase food security in the Pacific region [5]. Generally speaking these open ocean pelagic resources are under-utilised by coastal communities in the Pacific, the use of inshore fish aggregating devices (FADs) for sole use by subsistence communities shows great promise as a means to promote access to pelagic fisheries [5,33]. Understanding the social and biological drivers for changing fishing practices in Roviana through transitioning from inshore reef fishing to offshore pelagic fish may provide an important avenue for future research.

Previous research in the region [23,34] suggests that the annual fluctuation of species’ spatial and temporal distribution allows fishers to harvest numerous organisms at different times and places, with this variability being determined by lunar and tidal phases. Recurrent lunar aggregations are spatio-temporally predictable occurrences that can increase a fisher’s catches across various periods of the year. Sometimes fishers become specialists by targeting a limited number of species, while at others times they act as generalists and exploit a wide range of species in various marine habitats.

In summary, the results presented in this paper concur with regional scale generalisations that the productivity of inshore fisheries maybe declining as population growth outpaces supply, as evidenced by the fall in catch rates between 1995 and 2011 observed in this study. However the simplistic models that this linear supply and demand equation will lead to a depletion of inshore fisheries and hence severe food security issues for many Pacific nations does not fully consider the behavioural plasticity of Pacific communities allowing them to adapt to environmental change. This study provides evidence that increasing access and utilisation of offshore pelagic fish resources will likely play a critical role in food security as reef fish resources decline. In addition the high reliance of many Melanesian communities on mangrove resources such as crabs and bivalves [35,36] provides further buffering and adaptive capacity to the projected declines in reef fisheries.

## Supporting Information

S1 Fig Map of Solomon Islands and study location. (TIF)

S1 Table Statistical results of Kruskal-Wallis analysis by fishing method. (DOCX)

## References

[1] FAO (2014) The State of World Fisheries and Aquaculture. Rome: Food and Agriculture Organisation of the United Nations.

[2] Pauly D (2007) The Sea Around Us Project: Documenting and Communicating Global Fisheries Impacts on Marine Ecosystems. AMBIO 36: 290–295. 17626465 10.1579/0044-7447(2007)36[290:tsaupd]2.0.co;2

[3] Pauly D (2008) Global Fisheries: A Brief Review. J Biol Res (Thessalon) 9: 3–9.

[4] Hall SJ, Hilborn R, Andrew NL, Allison EH (2013) Innovations in capture fisheries are an imperative for nutrition security in the developing world. Proc Natl Acad Sci U S A 110: 8393–8398. 10.1073/pnas.1208067110 23671089 PMC3666725

[5] Bell JD, Allain V, Allison EH, Andréfouët S, Andrew NL, Batty MJ et al (2015) Diversifying the use of tuna to improve food security and public health in Pacific Island countries and territories. Marine Policy 51: 584–591.

[6] Bell JD, Kronen M, Vunisea A, Nash WJ, Keeble G, Demmke A, et al (2009) Planning the use of fish for food security in the Pacific. Marine Policy 33: 64–76.

[7] Bell J, Reid C, Batty M, Lehodey P, Rodwell L, Hobday A, et al (2013) Effects of climate change on oceanic fisheries in the tropical Pacific: implications for economic development and food security. Clim. Change 119: 199–212.

[8] Bell JD, Ganachaud A, Gehrke PC, Griffiths SP, Hobday AJ, et al (2013) Mixed responses of tropical Pacific fisheries and aquaculture to climate change. Nat Clim Chang 3: 591–599.

[9] Gillett R, Cartwright I (2010) The future of Pacific Island fisheries. Noumea, New Caledonia: Secretariat of the Pacific Community.

[10] SPC (2013) Status Report: Pacific Islands reef and nearshore fisheries and aquaculture. Noumea, New Caledonia: Secretariat of the Pacific Community.

[11] Harley S, Williams P, Nicol S, Hampton J (2014) The western and central pacific tuna fishery: 2012 Overview and status of stocks. Noumea, New Caledonia: Secretariat of the Pacific Community.

[12] Gillett R (2009) Fisheries and the economies of the Pacific Island Countries and Territories. Mandaluyong City, Phitippines: Asian Development Bank.

[13] Richards AH, Bell LJ, Bell JD (1994) Inshore fisheries resources of Solomon Islands. Mar Pollut Bull 29: 90–98.

[14] Dalzell P, Adams TJH (1996) Sustainability and Management of Reef Fisheries in the Pacific Islands. 8th International Coral Reef Symposium Panama City.

[15] Kuster C, Vuki VC, Zann LP (2005) Long-term trends in subsistence fishing patterns and coral reef fisheries yield from a remote Fijian island. Fish Res 76: 221–228.

[16] Craig P, Green A, Tuilagi F (2008) Subsistence harvest of coral reef resources in the outer islands of American Samoa: Modern, historic and prehistoric catches. Fish Res 89: 230–240.

[17] Aswani S, Sabetian A (2010) Implications of Urbanization for Artisanal Parrotfish Fisheries in the Western Solomon Islands. Conserv Biol 24: 520–530. 10.1111/j.1523-1739.2009.01377.x 19961509

[18] Brewer TD, Cinner JE, Fisher R, Green A, Wilson SK (2012) Market access, population density, and socioeconomic development explain diversity and functional group biomass of coral reef fish assemblages. Glob Environ Change 22: 399–406.

[19] Brewer TD, Cinner JE, Green A, Pandolfi JM (2009) Thresholds and multiple scale interaction of environment, resource use, and market proximity on reef fishery resources in the Solomon Islands. Biol Conserv 142: 1797–1807.

[20] Adams TJH, Dalzell P, Farman R (1996) Status of Pacific Island Coral Reef Fisheries. 8th International Coral Reef Symposium Panama.

[21] Dalzell P, Adams TJH, Polunin NVC (1996) Coastal fisheries in the Pacific Islands. Oceanography and Marine Biology: An Annual Review 34: 395–531.

[22] Skewes T (1990) Marine Resource Profiles; Solomon Islands. Honiara, Solomon Islands: Pacific Islands Forum Fisheries Agency. 59 p.

[23] Aswani S, Lauer M (2014) Indigenous People's Detection of Rapid Ecological Change. Conserv Biol 28: 820–828. 10.1111/cobi.12250 24528101

[24] Lauer M, Aswani S (2010) Indigenous Knowledge and Long-term Ecological Change: Detection, Interpretation, and Responses to Changing Ecological Conditions in Pacific Island Communities. Environ Manage 45: 985–997. 10.1007/s00267-010-9471-9 20336296 PMC2871288

[25] Aswani S., 1997 Customary sea tenure and artisanal fishing in the Roviana and Vonavona Lagoons, Solomon Islands: the evolutionary ecology of marine resource utilization, Department of Anthropology. University of Hawaii, p. 485.

[26] Halpern BS, Selkoe KA, White C, Albert S, Aswani S, Lauer M (2013) Marine protected areas and resilience to sedimentation in the Solomon Islands. Coral Reefs 32: 61–69.

[27] Aswani S (1998) Patterns of marine harvest effort in southwestern New Georgia, Solomon Islands: resource management or optimal foraging? Ocean Coast Manag 40: 207–235.

[28] Albert S, Dunbabin M, Skinner M, Moore B, Grinham A (2012). Benthic Shift in a Solomon Island's lagoon: corals to cyanobacteria. 12th International Coral Reef Symposium, Australia.

[29] Lauer M, Albert S, Aswani S, Halpern BS, Campanella L, La Rose D (2013) Globalization, Pacific Islands, and the paradox of resilience. Glob Environ Change 23: 40–50.

[30] Friedlander AM, DeMartini EE (2002) Contrasts in density, size, and biomass of reef fishes between the northwestern and the main Hawaiian islands: the effects of fishing down apex predators. Mar Ecol Prog Ser 230: 253–264.

[31] Newton K, Côté IM, Pilling GM, Jennings S, Dulvy NK (2007) Current and Future Sustainability of Island Coral Reef Fisheries. Curr Biol 17: 655–658. 17382547 10.1016/j.cub.2007.02.054

[32] Anon. (2009) Solomon Island Population and Housing Census 2009. Solomon Islands Government.

[33] Albert JA, Beare D, Schwarz AM, Albert S, Warren R, Teri J, et al. (In press) The contribution of nearshore fish aggregating devices (FADs) to food security and livelihoods in Solomon Islands. PLOS One. 10.1371/journal.pone.0115386 PMC4267842 25513808

[34] Aswani S, Vaccaro I (2008) Lagoon Ecology and Social Strategies: Habitat Diversity and Ethnobiology. Hum Ecol 36: 325–341.

[35] Aswani S, Flores C, Broitman B. (2015) Human harvesting impacts on managed areas: Ecological effects of socially-compatible shellfish reserves. Rev of Fish Biol Fish 25: 217–230.

[36] Grinham A, Kvennefors C, Fisher PL, Gibbes B, Albert S (2014) Baseline arsenic levels in marine and terrestrial resources from a pristine environment: Isabel Island, Solomon Islands. Mar Pollut Bull. 88: 354–360. 10.1016/j.marpolbul.2014.08.018 25199709

