# Supplementary figures and images for: Keeping Food on the Table: Human Responses and Changing Coastal Fisheries in Solomon Islands
Source: PLoS One. 2015 Jul 9;10(7):e0130800. doi: 10.1371/journal.pone.0130800 (PMC4497618; doi:10.1371/journal.pone.0130800)

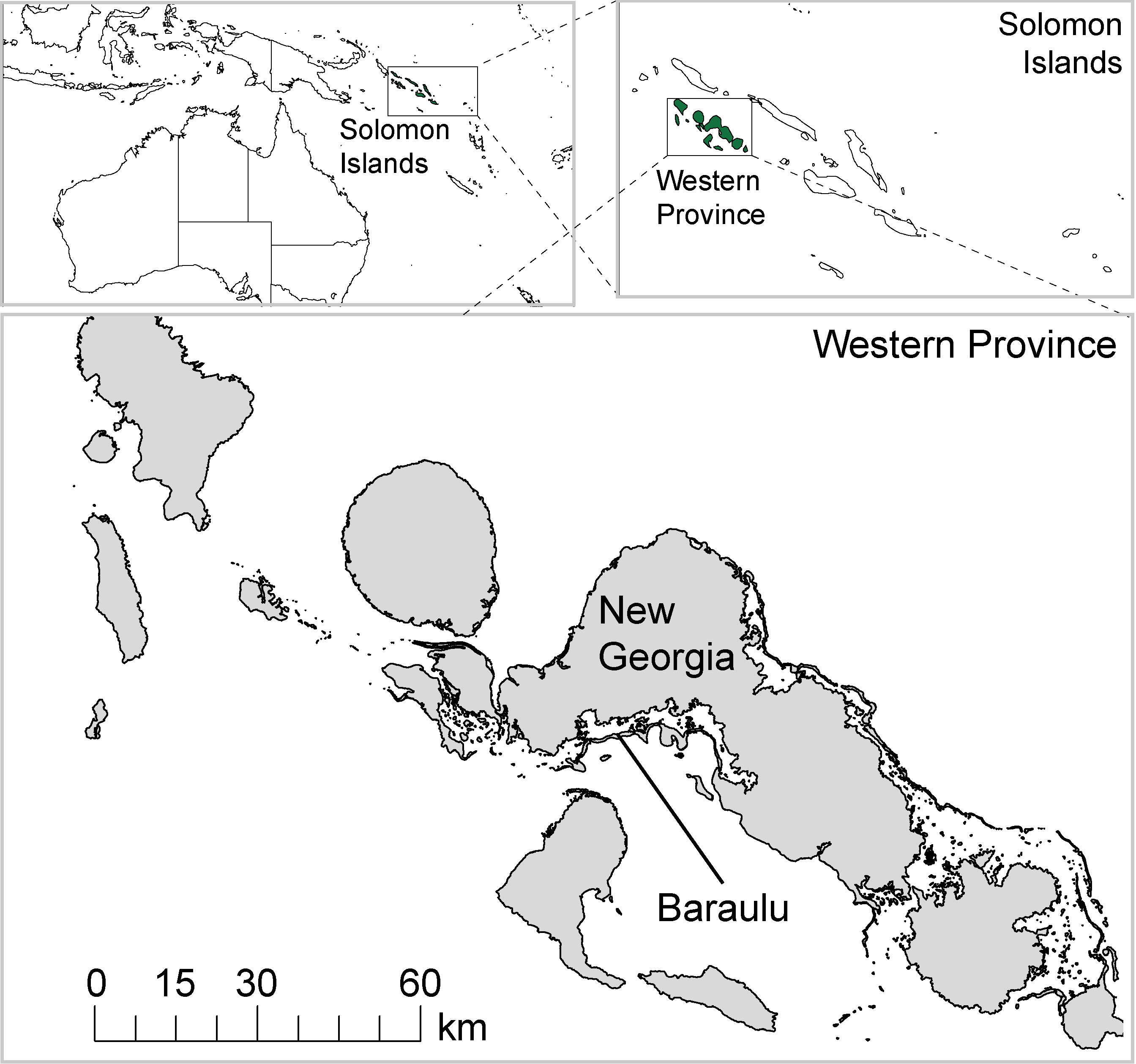

Supplement: S1 Fig — (TIF) [file pone.0130800.s001.tif]
