# Supplementary material for: Keeping Food on the Table: Human Responses and Changing Coastal Fisheries in Solomon Islands
Source: PLoS One. 2015 Jul 9;10(7):e0130800. doi: 10.1371/journal.pone.0130800 (PMC4497618; doi:10.1371/journal.pone.0130800)
Supplement: S1 Table — (DOCX) [file pone.0130800.s002.docx]

**S1 Table 1. Statistical results of Kruskal-Wallis analysis by fishing method.**

| **Fishing Method** | **Parameter** | **Statistics** |
| --- | --- | --- |
| Dropline | Mean fishing time | KW_(1,79)_ = 26.24 p < 0.001 |
|  | Mean distance travelled | KW_(1,79)_ = 7.46 p = 0.006 |
|  | Mean catch weight | KW_(1,79)_ = 13.55 p < 0.001 |
|  | Mean fish size | KW_(1,79)_ = 3.52 p = 0.06 |
|  | CPUE | KW_(1,79)_ = 1.37 p = 0.24 |
| Handline | Mean fishing time | KW_(1,662)_ = 203.73 p < 0.001 |
|  | Mean distance travelled | KW_(1,662)_ = 38.36 p < 0.001 |
|  | Mean catch weight | KW_(1,662)_ = 40.63 p < 0.001 |
|  | Mean fish size | KW_(1,641)_ = 46.67 p < 0.001 |
|  | CPUE | KW_(1,662)_ = 30.89 p < 0.001 |
| Net | Mean fishing time | KW_(1,101)_ = 22.51 p < 0.001 |
|  | Mean distance travelled | KW_(1,101)_ = 0.22 p = 0.63 |
|  | Mean catch weight | KW_(1,101)_ = 8.64 p = 0.003 |
|  | Mean fish size | KW_(1,80)_ = 0.01 p = 0.93 |
|  | CPUE | KW_(1,101)_ = 0.58 p < 0.46 |
| Trolling | Mean fishing time | KW_(1,146)_ = 59.43 p < 0.001 |
|  | Mean distance travelled | KW_(1,146)_ = 50.52 p < 0.001 |
|  | Mean catch weight | KW_(1,146)_ = 11.16 p < 0.001 |
|  | Mean fish size | KW_(1,145)_ = 6.98 p = 0.008 |
|  | CPUE | KW_(1,146)_ = 5.83 p = 0.015 |
